# Evaluation of the Siemens Healthineers Atellica TSTII testosterone assay in the CDC HoSt-TT program

**DOI:** 10.1210/clinem/dgag071

**Published:** 2026-02-19

**Authors:** Jieli Li, Scott D Isaacs, Samantha Pagliaro, Usman Sunusi, JoDell E Wilson, Edward B De Vol, Ross Molinaro, Neil Parker, Jeffrey I Mechanick

**Affiliations:** Department of Pathology, The Ohio State University College of Medicine, Columbus, OH 43210, USA; Department of Medicine, Emory University School of Medicine, Atlanta, GA 30322, USA; Department of R&D, Siemens Healthineers, Tarrytown, NY 10591, USA; Lake Erie College of Osteopathic Medicine, School of Dental Medicine, Bradenton, FL 34211, USA; Department of Medical Affairs, Siemens Healthineers, Tarrytown, NY 10591, USA; Department of Medical Affairs, Siemens Healthineers, Tarrytown, NY 10591, USA; Department of Medical Affairs, Siemens Healthineers, Tarrytown, NY 10591, USA; Department of R&D, Siemens Healthineers, Tarrytown, NY 10591, USA; Mount Sinai Fuster Heart Hospital, Icahn School of Medicine at Mount Sinai, NewYork City, NY 10029, USA

**Keywords:** total testosterone, free testosterone, hypogonadism, CDC hoSt-TT, atellica IM & CI TSTII, LC-MS/MS

## Abstract

**Background:**

Standardized testosterone measurement is essential for diagnosing hypogonadism, infertility, endocrine disorders, and monitoring therapy. The CDC Hormone Standardization Program for Total Testosterone (HoSt-TT) evaluates assay performance against ID-LC-MS/MS using clinically relevant concentrations. This study assesses the Atellica IM TSTII assay compliance to HoSt-TT criteria and its correlation with LC-MS/MS.

**Methods:**

Analytical performance of Atellica IM TSTII and method comparison studies using HoSt-TT certification samples were analyzed, evaluated, and stratified by sex. Comparisons of TSTII TT to LC-MS/MS and calculated vs measured free testosterone (FT) were performed and analyzed using remnant patient samples, with FT calculated (Vermeulen method, ISSAM calculator).

**Results:**

HoSt-TT vs Atellica IM TSTII assay results demonstrated average bias within ±6.4%, with OLS regression slopes of 1.02-1.06, *r* ≥ 0.98. TSTII demonstrated repeatability (2.18% CV), within-lab precision (3.46% CV), and overall total analytical error of 18.3% (IQR 11.6-31.6%). Furthermore, comparing TT on the TSTII to LC-MS/MS (LDT) using native patient samples, resulted in strong fits (slope = 0.9798, *r* = 0.991); and calculated FT correlated well with equilibrium dialysis/LC-MS/MS (slope = 0.9530, *r* = 0.965).

**Conclusion:**

The TSTII assay is CDC HoSt-TT certified, has strong alignment to LC-MS/MS, and shows good correlation of calculated FT with equilibrium dialysis/LC-MS/MS. Although LC-MS/MS remains the reference method for testosterone quantification, particularly at low concentrations, CDC HoSt certification of the TSTII assay supports its potential suitability for measuring testosterone concentrations in women and children within defined analytical and clinical contexts.

Testosterone is a key steroid hormone with essential roles in sexual development, reproductive function, musculoskeletal health, and bone metabolism. It is synthesized predominantly by the testes in males, with smaller contributions from the ovaries in females and the adrenal glands in both males and females. Accurate quantification of testosterone across diverse populations, including men, women, children, and transgender individuals, is clinically important, as abnormal levels are associated with a wide range of conditions such as hypogonadism, polycystic ovary syndrome, ovarian and adrenal tumors, congenital adrenal hyperplasia, and disorders of puberty. Additional indications for testosterone measurement include evaluation of unexplained anemia, osteoporosis, type 2 diabetes, infertility, chronic opioid use, HIV infection, prior exposure to chemotherapy or radiotherapy, and forensics (1, 2). Formalized evidence-based recommendations governing the use of total testosterone (TT) and free testosterone (FT) levels for the diagnosis and management of these disorders are provided by several clinical practice guidelines. Various analyte thresholds are specified such as for a diagnosis of hypogonadism with TT levels < 300 ng/dL (American Urological Association [AUA] (3, 4)) or < 264 ng/dL (1, 5) depending on the society or study referenced. The Endocrine Society, supporting the latter cutoff, recommends repeating morning TT measurements due to the diurnal variation of testosterone levels resulting in the highest levels during the early morning (1). The guidelines also support the use of free testosterone testing when TT levels are borderline or when alterations in sex hormone-binding globulin (SHBG) are suspected (1). While TT is the primary marker used in most clinical settings, its utility depends heavily on assay accuracy, particularly at decision thresholds that guide diagnosis and treatment. Direct immunoassays, though widely used, are susceptible to interference and lack harmonization, leading to variability in results. More labor-intensive methods such as LC-MS/MS can offer superior accuracy and sensitivity but are less practical for routine use (6-8). Even small variations in assay performance can lead to misclassification of patients, inappropriate therapy, or missed diagnoses. For this reason, standardization of TT measurement techniques is essential to ensure reliable interpretation and consistent clinical decision-making.

Achieving standardization of testosterone testing, however, remains a significant challenge. Inter-assay variability and inconsistent calibration across laboratories and platforms limit comparability of results, especially at low concentrations where performance differences between immunoassays and LC-MS/MS are most noticeable. These discrepancies continue to pose barriers to consistent interpretation and guideline-based clinical care.

To address these concerns, the Centers for Disease Control and Prevention (CDC) launched the Hormone Standardization Program for Testosterone (HoSt-TT) in 2010. The program provides reference materials and a traceability framework aligned to the CDC's reference measurement procedure (RMP) using isotope dilution LC-MS/MS (ID-LC-MS/MS). Certification through the HoSt-TT program ensures traceability, minimizes variability, and supports compliance with Endocrine Society and AUA guidelines (1, 4). For laboratories and clinicians, immunoassays that achieve and maintain this certification provide confidence in the reliability of results, particularly given that immunoassays remain the most widely used method in routine practice, while LC-MS/MS is less practical outside specialized settings. This is especially important when TT is used to calculate free or bioavailable fractions where small inaccuracies can affect clinical interpretation. Persistent challenges in testosterone assay harmonization have been demonstrated in large interlaboratory surveys. Cao et al evaluated total testosterone performance among New York State–approved laboratories in 2017 and again approximately 3.5 years later, showing that both immunoassays and LC-MS/MS methods continued to exhibit clinically meaningful variability over time. Importantly, assays that maintained CDC HoSt-TT certification demonstrated more stable alignment with reference targets, whereas several non-certified assays drifted outside acceptable performance limits during follow-up (9, 10). These findings underscore the importance of ongoing standardization efforts and external certification programs such as HoSt-TT to ensure longitudinal consistency and reliable clinical interpretation of testosterone results.

This study evaluated the long-term alignment of Siemens Healthineers TSTII immunoassay on the Atellica IM and Atellica CI Analyzers with the CDC HoSt-TT program. In addition, we reported on method comparison, bias, within-lab precision (between day), and total analytical error (TAE) to support the clinical value of using the CDC-certified Siemens Healthineers TSTII immunoassay.

## Materials and methods

### Siemens Healthineers atellica IM Total Testosterone (TSTII) assay procedures

The Siemens Healthineers Atellica IM TSTII assay on the Atellica IM and Atellica CI Analyzers is standardized using internal standards made from United States Pharmacopeia (USP)-grade testosterone (11, 12). The internal standards are traceable to the CDC HoSt ID-LC-MS/MS RMP using comparisons of value-assigned samples (13). The ID-LC-MS/MS RMP is traceable to the primary total testosterone standard M914 from the Australian National Measurement Institute (A-NMI) (14).

The Atellica IM TSTII assay is a 14-minute quantitative competitive immunoassay that employs paramagnetic particles and chemiluminescent technology and is intended for use in the quantitative determination of TT in human serum and plasma. This TSTII assay consists of a releasing agent to displace testosterone from its binding protein, a biotin-labeled anti-testosterone sheep monoclonal antibody as the capture conjugate, streptavidin magnetic latex particles as the solid phase, and an acridinium ester-labeled hapten conjugate as the Lite reagent. Testosterone in the patient sample competes with the acridinium ester-labeled hapten for binding with the anti-testosterone.

### HoSt-TT program certification

Materials used in the HoSt-TT Program are unaltered human sera from single donors, self-reporting sex, obtained following the Clinical and Laboratory Standards Institute guideline C37-A protocol (15). Donor demographic information regarding sex was provided by the CDC HoSt-TT program and was not independently verified by the investigators. Sex designation was used only for sample description and was not used to interpret testosterone concentrations or establish clinical reference thresholds. ID-LC-MS/MS was used with electrospray ionization in the positive ion mode monitoring the 289→97 m/z (testosterone) and 292→112 m/z (^3^C-13 testosterone) and certified primary standards from the A-NMI (16). The CDC RMP for testosterone has been recognized by the Joint Committee for Traceability in Laboratory Medicine as a higher-order reference method, based on comparison studies with established reference procedures at the National Institute of Standards and Technology and the University of Ghent (13, 16, 17). Therefore, these materials are traceable, as described in the International Organization for Standardization 17511, to A-NMI M914.

For HoSt-TT certification, four quarterly challenges were provided, each with 10 blinded human serum samples and tested in duplicate across two days (*n* = 4 results/sample). Certification is granted if the average bias across all 40 samples (10 samples across 4 quarters) remains within ±6.4% of CDC assigned values (17).

### Alignment to CDC HoSt RMP for total testosterone

The Siemens Healthineers Atellica IM TSTII assay has maintained CDC HoSt-TT certification since 2019, and the Atellica CI analyzer achieved certification using the same assay in 2024 (18). To date, Siemens Healthineers assays remain the only total testosterone immunoassays reported to be CDC HoSt-TT certified (18).

For this study, data from 24 quarterly shipments (2019-2024) were evaluated. Bias was calculated as the difference between the 4-replicate mean and the CDC-assigned value, while precision was assessed as repeatability (within-run CV) and within-lab precision (between-day CV). A multi-level (run nested within days) model accounted for variability among replicates. Rolling four-quarter averages of bias, repeatability, and within-lab precision were plotted over time. In this study, TAE was estimated by combining observed bias with within-lab precision, thereby reflecting both systematic and random components of assay performance. This approach provides an empirical measure of assay error and should be distinguished from total allowable error (TEa), which represents a predefined performance goal or quality specification.

### Independent method comparison

An independent method comparison of TT was conducted using 24 remnant samples. Analytical comparisons were performed using de-identified residual patient samples; The Ohio State University IRB approval was waived. TT was measured by two CDC HoSt-TT certified methods: the Atellica IM TSTII assay (testing performed at The Ohio State University Clinical Laboratory) and LC-MS/MS total testosterone assay (testing performed by Mayo Clinical Laboratories). Correlation between the two methods was assessed using ordinary least squares (OLS) regression.

FT comparisons were conducted using 25 remnant patient samples. FT was measured using Equilibrium Dialysis/LC-MS/MS (Mayo Clinical Laboratory) and calculated using values obtained from testing with immunoassays. For the calculated FT, TT (Atellica IM TSTII), and SHBG (Atellica IM SHBG) were assayed on the Atellica IM Analyzer at The Ohio State University Clinical Laboratory. FT was then calculated using the Vermeulen et al method via the International Society for the Study of the Aging Male (ISSAM) free testosterone calculator, based on measured TT and SHBG concentrations (19, 20). Calculated FT results were compared with those obtained by equilibrium dialysis/LC-MS/MS. OLS and Bland–Altman plots were used to evaluate agreement.

## Results

### HoSt-TT certification and long-term performance of Siemens Healthineers TSTII assay

**Figure 1:**
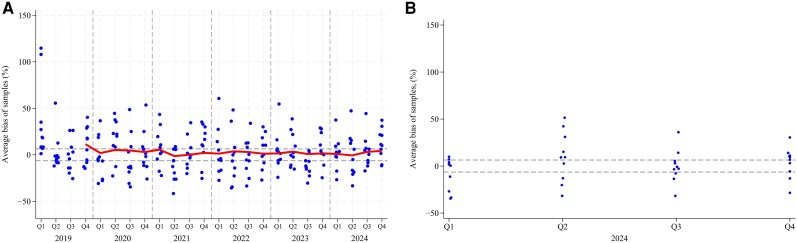
Long-term bias performance of Siemens Healthineers Atellica TSTII assays compared with CDC HoSt-TT reference values. (A) Quarterly average bias of Atellica IM TSTII results from 2019-2024 (*n* = 240). (B) Quarterly average bias of Atellica CI TSTII results during 2024. Horizontal dashed lines indicate HoSt-TT allowable bias limits (±6.4%).

Longitudinal performance of the Atellica IM TSTII assay (Fig. 1A) showed stable agreement with CDC HoSt-TT reference values across 24 quarters (2019-2024). After Q1 2019 assessment, a calibration adjustment was introduced to improve alignment at lower testosterone concentrations. From Q2 2019 forward, quarterly mean biases consistently fell within the HoSt-TT allowable limits (±6.4%), supporting sustained certification. Evaluation of the Atellica CI analyzer in 2024 (Fig. 1B) demonstrated similar performance, with all quarterly results remaining within certification criteria. Together, these findings confirm that both Atellica platforms maintain long-term accuracy and compliance with CDC HoSt-TT standards

### HoSt-TT sample distribution and precision

**Figure 2:**
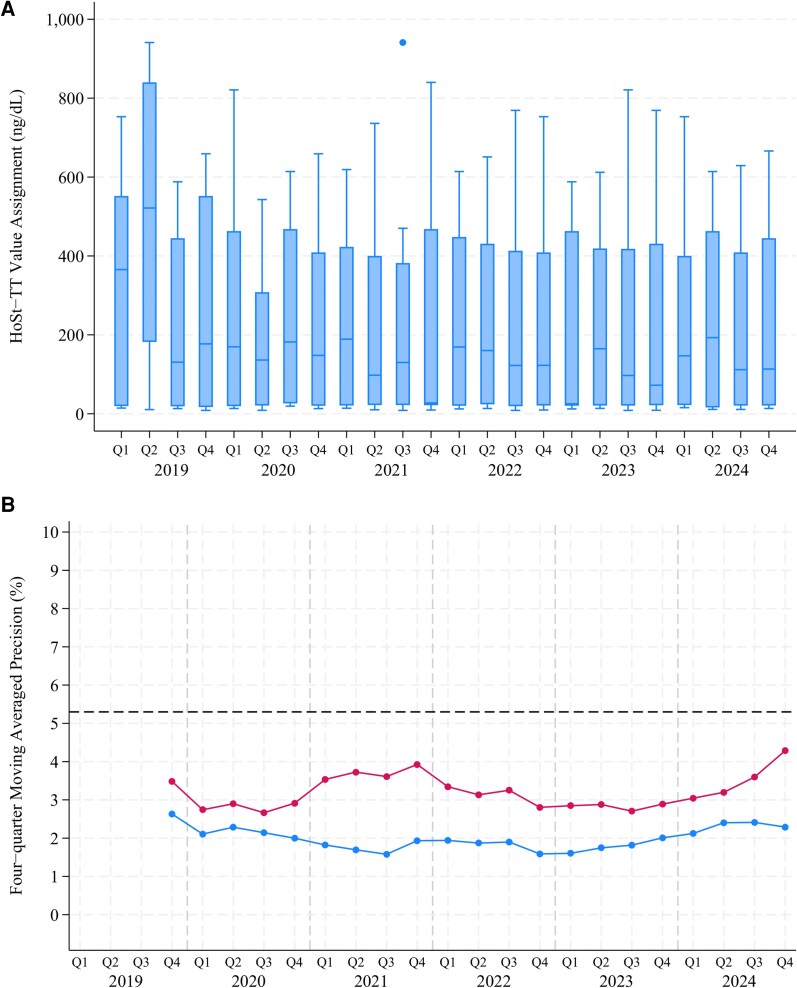
Distribution of HoSt-TT challenge samples and long-term precision performance of the Atellica IM TSTII assay. (A) Boxplots of CDC-assigned TT concentrations (*n* = 240) distributed for HoSt-TT certification from 2019- to 2024. In 22 of 24 quarters, median TT values were below 200 ng/dL, with occasionally higher concentrations included to extend the measurement range. Overall, sample distribution was balanced between male and female donors, though sex is not shown in this plot. (B) Four-quarter moving averages of repeatability (lower curve) and within-laboratory precision (upper curve) for the Atellica IM TSTII assay across 24 quarters (2019-2024). Both measures remained stable and consistently below the HoSt-TT maximum allowable imprecision threshold of 5.3% (dashed line). Note, two samples Reading above 100% bias were from 2019 Q1, prior to restandardization of the TSTII assay.

Across 24 quarters of HoSt-TT challenges (2019-2024), the CDC distributed samples spanned a wide range of TT concentrations, with a strong emphasis on the lower end of the male clinical spectrum. In 22 of 24 quarters, median TT values were below 200 ng/dL, reflecting the program's focus on concentrations near decision thresholds for male hypogonadism. Occasional inclusion of higher concentrations ensured adequate coverage across the clinically relevant measurement range. The distribution of samples was approximately balanced between male and female donors, providing a representative assessment of assay performance (Fig. 2A).

Longitudinal precision analysis of the Atellica IM TSTII assay, expressed as four-quarter moving averages, showed consistent repeatability (within the manufacturer's claims: ≤ 2.7 SD or ∼2%CV (<15 ng/dL), ≤ 18%CV (15—20 ng/dL); ≤ 8%CV (20—1000 ng/dL), and ≤ 11%CV (≥1000 ng/dL) (21)) and within-lab precision (∼3-4% CV) throughout the six-year study period. Both measures remained well below the HoSt-TT recommended imprecision limit of 5.3%, confirming stable assay performance over time and supporting the assay's reliability in sustained clinical use (Fig. 2B).

### Annual assessment of alignment with the HoSt-TT RMP

**Figure 3:**
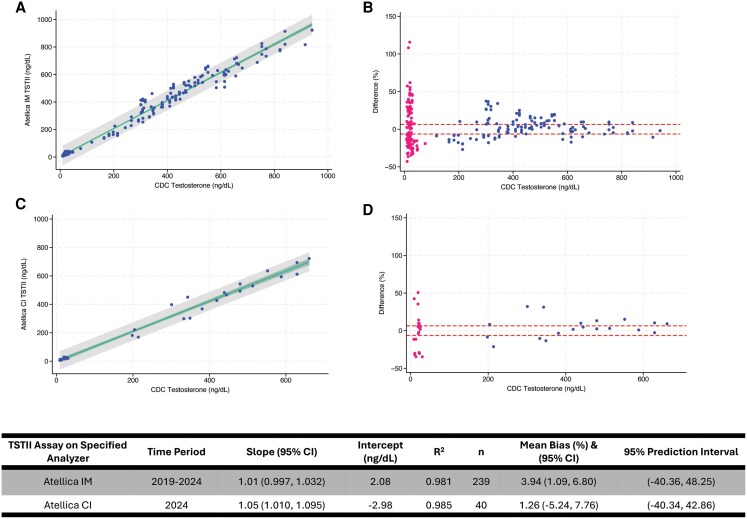
Ordinary least squares linear fits and Bland–Altman difference plots of atellica IM TSTII vs CDC HoSt program-TT value-assigned TT samples. TT concentrations (ng/dL) were calculated as the mean of four measurements according to CDC HoSt-TT certification protocol. Results are shown for TSTII assays on: (A,B) Atellica IM (*n* = 240), (C,D) Atellica CI (*n* = 40). Panels A and C shows OLS fits with light gray bands representing 95% prediction intervals, highlighting the narrow distribution. Panels B and D present Bland-Altman difference plots with dashed lines indicating ±6.4% difference thresholds; whereas, pink dots represent female results and blue dots represent male results.

Comparison of Atellica TSTII results with CDC-assigned reference values demonstrated strong agreement for both the Atellica IM (2019-2024) and Atellica CI (2024) platforms. Ordinary least-squares regression showed slopes close to unity (1.01 for Atellica IM and 1.05 for Atellica CI) with negligible intercepts and large coefficients of determination (*R*² ≥ 0.98), indicating strong linear association across the full concentration range (Fig. 3A, C). Mean biases were modest, 3.9% for Atellica IM and 1.3% for Atellica CI, both within the HoSt-TT acceptance criteria. Percent difference plots (Fig. 3B, D) revealed greater variability at the lowest concentrations, but no evidence of systematic deviation across the measurement range (Fig. 3A). Together, these findings confirm consistent alignment of Siemens Healthineers TSTII assays with CDC reference values over multiple years of certification.

**Figure 4:**
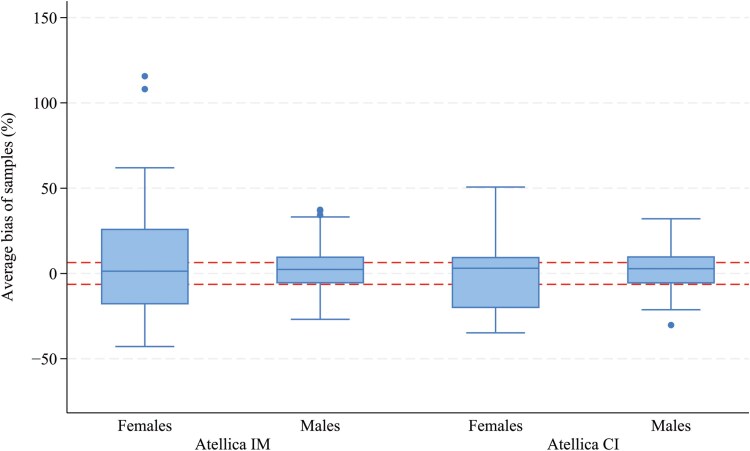
Sex-stratified distribution of average sample bias for Atellica IM and Atellica CI TSTII assays. Boxplots show the distribution of average bias (%) for samples from males and females analyzed on the Atellica IM (left, *n* = 240) and Atellica CI (right, *n* = 40) platforms, relative to CDC HoSt-TT reference values. Red dashed lines represent the HoSt-TT allowable bias limits of ±6.4%. Median biases for both males and females remained within acceptance limits on both platforms, with wider variability observed among samples from females due to lower testosterone concentrations.

Figure 4 illustrates the distribution of average sample bias stratified by sex for both the Atellica IM and Atellica CI TSTII assays, relative to CDC HoSt-TT reference values. Median biases for samples from both males and females were within the HoSt-TT acceptance limits (±6.4%), confirming overall alignment with reference standards. As expected, variability was greater among samples from females due to lower testosterone concentrations, but no systematic sex-related bias was observed on either platform.

### Atellica IM TSTII: precision and total analytical error overall and by sex

**Figure 5:**
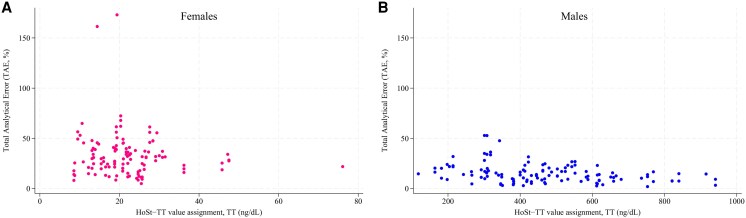
Total analytical error (TAE) of the Atellica IM TSTII assay by sex across CDC HoSt-TT challenge samples. Panels show the distribution of TAE as a function of testosterone concentration in females (left, *n* = 118) and males (right, *n* = 122). Variability was greater at lower concentrations typical of females, while TAE values in males were generally lower and more stable across the measurement range.

Precision of the Atellica IM TSTII assay was evaluated using HoSt-TT blind sample challenge data. Across all quarters, the assay demonstrated repeatability of 2.1% CV and within-lab precision of 2.0% CV and reproducibility of 3.4% CV when run on the Atellica IM Analyzer (Table 1). When stratified by sex, TAE was notably higher in samples from females, which represent lower testosterone concentrations, compared to samples from males with higher concentrations (Fig. 5). The median TAE was 29.7% for females and 13.5% for males. Samples from females also showed greater variability, with several results exceeding 100% TAE at very low concentrations. In contrast, samples from males demonstrated tighter clustering around lower TAE values across a wide concentration range. These findings highlight the analytical complexity of accurately measuring testosterone at low level concentrations typically observed in females.

**Table 1:** Precision of the Atellica IM TSTII assay on Atellica IM and Atellica CI analyzers determined using HoSt-TT materials

Atellica IM		Repeatability (SD)	Repeatability (% CV)	Within-Lab or Between Day (SD)	Within-Lab or Between Day (% CV)	Reproducibility (SD)	Reproducibility (% CV)
Overall		4.2	2.1	3.7	2.0	6.7	3.4
2019	Q1	9.1	4.5	6.6	2.3	13.6	5.7
	Q2	10.4	1.6	1.5	0.8	11.0	2.2
	Q3	6.0	2.5	1.3	2.0	6.6	3.5
	Q4	5.1	1.9	3.4	1.1	7.0	2.5
2020	Q1	5.4	2.4	1.4	0.8	6.1	2.7
	Q2	3.7	2.4	0.9	0.8	4.1	2.8
	Q3	3.0	1.9	3.3	1.1	5.4	2.6
	Q4	2.6	1.4	5.2	3.0	6.1	3.5
2021	Q1	2.6	1.7	11.8	4.7	12.2	5.2
	Q2	4.3	1.9	3.0	2.5	5.7	3.6
	Q3	3.0	1.4	1.5	1.2	3.8	2.1
	Q4	5.1	2.8	7.9	3.1	10.2	4.8
2022	Q1	4.3	1.7	1.7	1.8	5.0	2.9
	Q2	2.4	1.6	2.7	1.8	3.8	2.7
	Q3	3.2	1.5	2.8	1.7	5.1	2.6
	Q4	2.7	1.5	1.9	2.0	4.0	3.0
2023	Q1	2.6	1.8	1.7	1.8	3.5	3.1
	Q2	2.3	2.2	2.1	1.3	3.7	2.8
	Q3	2.7	1.8	0.5	0.3	2.9	1.9
	Q4	5.0	2.3	9.1	2.5	10.9	3.7
2024	Q1	4.0	2.2	2.7	2.4	5.7	3.7
	Q2	4.9	3.3	0.6	0.6	5.2	3.5
	Q3	3.4	1.8	3.5	2.2	5.8	3.5
	Q4	3.7	1.8	11.7	6.1	12.5	6.5

CI		Repeatability (SD)	Repeatability (% CV)	Between day (SD)	Between day (% CV)	Reproducibility (SD)	Reproducibility (% CV)
2024	Q1	3.9	2.30	0.9	0.40	4.2	2.5
	Q2	4.6	2.90	3.1	1.70	6.1	3.8
	Q3	1.4	1.00	2.7	1.20	3.6	1.9
	Q4	6.0	3.20	2.3	1.00	7.1	3.7

*n* = 10 samples/quarter, 40/year.

Within-lab precision of the Atellica IM TSTII assay varied across concentration ranges, with CVs generally remaining below 5%. At very low concentrations (<20 ng/dL), within-lab precision was lower (CVs 0-11%, or 3.2% on average) and TAE values were higher, reaching approximately 31.4% on average. In mid to higher concentration ranges (≥30 ng/dL), within-lab precision improved (CVs 0-6.6%, 1.4% on average), with TAE values of 15.9% on average. By contrast, the Atellica CI platform showed higher precision at the lowest concentrations (CVs 0-6.8%. or 2.0% on average; TAE of 36.7% on average). Precision at mid to higher TT ranges on the Atellica CI showed CVs of 0-4.0%, or 1.0% on average, with TAE of 15.4% on average. Data were not available for all ranges on the CI platform (Table 2).

**Table 2:** CLIA criteria pass rate, reproducibility, and total analytical error of the Atellica IM TSTII assay on the Atellica IM analyzer

TT (ng/dL)	Atellica IM		Atellica CI	
	Reproducibility, CV % average	TAE % average	Reproducibility, CV % average	TAE % average
0.0-8.9	4.2	15.7	7.2	26.0
9.0-20.0	5.4	38.7	3.9	38.1
20.1– 30.0	3.7	31.6	3.0	18.7
30.1-99.9	2.8	26.2	n/a	n/a
100.0-200.0	2.2	16.4	1.1	10.6
200.1-399.9	2.3	18.8	2.3	21.8
400.0-499.9	2.9	14.3	2.7	11.9
500.0-612.0	2.6	15.2	2.3	11.0
612.1-749.9	2.5	10.9	2.3	12.1
750.0-821.0	2.2	9.5	n/a	n/a
> 821	2.4	9.9	n/a	n/a

CLIA requires that total testosterone immunoassay results compare to proficiency testing within 20 ng/dL and/or 30%, TSTII performs at 100% in all but one range (200.1-399.9 ng/dL) showing greater than allowed bias of 0.03-13.2%. The median total analytical error (TAE) was estimated to be 19.6% (interquartile range 12.0% to 31.5%).

Concentration range of CDC HoSt Program-TT; samples provided for Q2 2019 through Q4 2024. Abbreviation: TT, total testosterone.

### External method comparison

**Figure 6:**
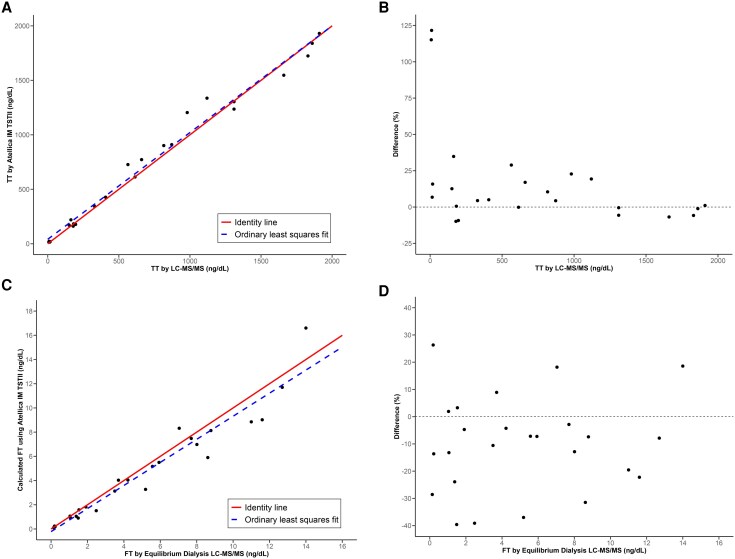
Method comparison of total testosterone (TT, *n* = 24) and calculated free testosterone (FT, *n* = 25) measured by the Siemens Healthineers Atellica IM TSTII assay vs LC-MS/MS reference methods. (A) OLS regression of TT measured by the Atellica IM TSTII assay compared with LC-MS/MS (Mayo Clinical Laboratories reference method). The regression equation (y = 0.98x + 41.1 ng/dL, *R*² = 0.982) demonstrates excellent linear agreement. The orange line represents the OLS fit, and the dashed line represents the line of identity. (B) Difference plot for TT showing percentage differences between Atellica IM TSTII and LC-MS/MS. Agreement was strong overall, with greater variability observed at lower TT concentrations. (C) OLS regression of calculated FT (derived from Atellica IM TSTII TT and SHBG using the ISSAM calculator) vs equilibrium dialysis LC-MS/MS. The regression equation (y = 0.953 × —0.22 ng/dL, *R*² = 0.931) indicates good agreement across the measured range, though a tendency toward underestimation at higher FT values is evident. (D) Difference plot for FT comparison, showing variability up to ±40% at lower concentrations. Despite this variability, overall trends were consistent, supporting clinical utility of calculated FT when TT and SHBG are standardized.

Comparison of total testosterone measurements between the Atellica IM TSTII and LC-MS/MS demonstrated strong agreement across the concentration range of 7.6-1910 ng/dL (*n* = 24). OLS regression yielded a slope of 0.98 with an intercept of +41.1 ng/dL (95% CI: ± 52 ng/dL) and *R*² of 0.982, indicating close alignment with the reference method and non-significant positive bias. The Bland–Altman analysis further showed that differences were generally small and evenly distributed across concentrations, with a few higher positive deviations observed at lower testosterone levels. Overall, these remnant patient sample results confirm that the Atellica IM TSTII assay provides highly comparable results to LC-MS/MS (Fig. 6 A&B). Calculated FT values using the Atellica IM TSTII and SHBG assays with the ISSAM calculator showed strong correlation with equilibrium dialysis (y = 0.953 × —0.22 ng/dL, *R*² = 0.931; *n* = 25). As shown in the regression plot, values aligned closely with the identity line across the measurement range, although a slight negative bias was observed. The Bland–Altman analysis confirmed this trend, demonstrating variability of up to ±40% at lower FT concentrations, while higher concentrations clustered closer to the reference method. These findings suggest that while calculated FT from the Atellica platform generally tracks with equilibrium dialysis, further studies are necessary to better understand correlation and interpretation at low concentrations, where discrepancies were observed (Fig. 6 C&D).

## Discussion

This study demonstrates that the Atellica IM TSTII assay maintains strong adherence to the CDC HoSt-TT certification criteria, achieving a total average bias within ±6.4% from Q1 2019 through Q4 2024 when run on the Atellica IM and CI Analyzer. Comparison with a HoSt-TT certified LC-MS/MS method shows excellent correlation, confirming that the TSTII immunoassay produces results aligned with the reference method across the clinically relevant testosterone range. Despite the ordinary scatter observed in immunoassays, these findings underscore the reliability and consistency of the TSTII assay in real-world laboratory settings and highlight the success of the HoSt-TT standardization program in ensuring accurate and harmonized TT measurements for patient care.

Male hypogonadism is defined by low TT levels; however, in some circumstances, assessment of bioavailable testosterone can alter classification of androgen status (eg, in obesity with low SHBG, where TT may be low but bioavailable testosterone remains normal). Since bioavailable testosterone is derived from TT values, accurate and standardized measurement of TT remains essential as the foundation for reliable interpretation of androgen status. The HoSt-TT blind sample challenge included materials spanning the clinically relevant testosterone range, with a substantial proportion of samples below the commonly used hypogonadism threshold of 264 ng/dL or 300 ng/dL cutoffs recommended by the Endocrine Society and AUA (eg, 303-853 ng/dL, central 90th percentile) (1, 4), respectively. For samples at this borderline clinical range (200-400 ng/dL), the Atellica IM TSTII assay demonstrated within-lab precision of 3.6%CV and TAE of 13.5%, reflecting precise and reliable assay performance. While CLIA defines total allowable error for testosterone as 20 ng/dL or 30%, which incorporates both analytical and biological variation, the observed TAE demonstrates that the assay's analytical performance is robust within this clinically important concentration range (22). Prior evaluations of testosterone assay standardization, including analyses by Cao et al, highlighted that acceptable average calibration bias did not necessarily translate to consistent agreement at the individual-sample level, particularly for earlier immunoassay platforms (10, 11). In contrast, the sustained HoSt-TT certification and per-sample performance observed for the Atellica TSTII assay in this study suggest meaningful improvements in both calibration and sample-level alignment over time.

To our knowledge, the TSTII assays on the Siemens Healthineers Atellica IM and Atellica CI analyzers are the only fully automated immunoassays to achieve and maintain CDC HoSt-TT certification (2019–present). The Atellica CI is the most recent platform to be certified, joining the HoSt-TT program in 2024. Standardization of hormone assays, particularly total testosterone, is critical to ensure that clinicians and laboratorians can interpret results accurately and consistently across platforms and institutions. Certification through programs such as HoSt-TT enables harmonized results, providing clinicians and patients with reliable data for informed medical decision-making. Standardized TT measurement also strengthens the clinical utility of immunoassays, supporting more accurate calculation of free testosterone across laboratories. The HoSt-TT program continues to demonstrate its value by providing reference methods and materials that ensure sensitive, reliable, and precise measurement of TT on Siemens Healthineers Atellica TSTII assays.

The analytical performance of immunoassay-based testosterone measurements has long been in question due previously observed poor analytical performance (20). However, at concentrations below approximately 50 ng/dL, where the majority of immunoassays may show increased imprecision and bias (9), the TSTII assay has shown remarkable precision and strong alignment under the CDC HoSt-TT program. For TT of 0.0-200.0 ng/dL measured by the TSTII assay, all measurements meet the CLIA criteria of acceptance (*n* = 477 on Atellica IM and *n* = 88 on the Atellica CI). The strong analytical performance at the low end (see Table S1 (23)) and through the entire clinical range of TT, has earned TSTII recognition as the one immunoassay remaining HoSt-TT CDC-certified (24). Because calculated free testosterone is mathematically derived from measured total testosterone, analytical imprecision or bias in total testosterone, particularly at low concentrations, can be amplified and affect the reliability of calculated values. While this study demonstrated good overall agreement between calculated and measured free testosterone, the limited number of samples at very low testosterone concentrations restricts conclusions regarding assay performance for free testosterone calculation in females and other low-concentration populations. Additional studies specifically designed to assess performance in these groups are warranted. The TSTII assay demonstrates excellent standardization and alignment with LC-MS/MS across the range assessed in this study and should be considered a reliable method for measuring testosterone in females, children, and patients requiring assessment at very low concentrations (21). In such settings, assay selection should be guided by the clinical context, required analytical sensitivity, and potential impact on patient management.

Although evaluation of between-laboratory variability was beyond the scope of this study, it remains an important consideration for clinical practice. Differences in calibration, assay implementation, and quality management across laboratories can contribute to variability in testosterone results, even when the same assay platform is used (5, 8, 25). Participation in standardization programs such as the CDC HoSt-TT is therefore critical, as it provides an external benchmark to minimize inter-laboratory variability and promote result comparability across institutions. Broader adoption of standardized assays and ongoing external quality assessment will be essential to further reduce between-laboratory variation and ensure consistent clinical interpretation of testosterone measurements.

Looking ahead, broader adoption of standardized testosterone assays has the potential to transform both clinical practice and laboratory medicine. While Siemens TSTII assays currently represent the only fully automated immunoassays with sustained HoSt-TT certification, extending similar standardization to other platforms will be essential to ensure equity and consistency of care across institutions. Future studies should further characterize assay performance in low-concentration populations, including females and pediatric patients, and define evidence-based thresholds for use of immunoassays and LC-MS/MS methods, alike. Beyond testosterone, the HoSt framework offers a model for harmonizing other hormone assays, where assay variability continues to limit clinical interpretation. Future research should also assess how the use of certified assays influences real-world outcomes, including diagnostic accuracy, treatment decisions, and cost-effectiveness. Ultimately, linking analytical standardization to patient outcomes will be critical in demonstrating the full value of programs such as HoSt-TT.

## Data Availability

All datasets generated during and/or analyzed during the current study are available from the corresponding author on reasonable request.

## Abbreviations

AUA: American Urological Association
CDC: Centers for Disease Control and Prevention
FT: free testosterone
OLS: ordinary least squares
RMP: reference measurement procedure
SHBG: sex hormone-binding globulin
TAE: total analytical error
TT: total testosterone
USP: United States Pharmacopeia

## References

[1] Bhasin S, Brito JP, Cunningham GR, et al Testosterone therapy in men with hypogonadism: an endocrine society clinical practice guideline. J Clin Endocrinol Metab. 2018;103(5):1715‐1744. 29562364 10.1210/jc.2018-00229

[2] Lood Y, Aardal E, Ahlner J, et al Determination of testosterone in serum and saliva by liquid chromatography-tandem mass spectrometry: an accurate and sensitive method applied on clinical and forensic samples. J Pharm Biomed Anal. 2021;195:113823. 33349473 10.1016/j.jpba.2020.113823

[3] Mulhall JP, Trost LW, Brannigan RE, et al Evaluation and management of testosterone deficiency, 2024. Accessed September 26, 2025. https://www.auanet.org/guidelines-and-quality/guidelines/testosterone-deficiency-guideline.html

[4] Mulhall JP, Trost LW, Brannigan RE, et al Evaluation and management of testosterone deficiency: AUA guideline. J Urol. 2018;200(2):423‐432. 29601923 10.1016/j.juro.2018.03.115

[5] Travison TG, Vesper HW, Orwoll E, et al Harmonized reference ranges for circulating testosterone levels in men of four cohort studies in the United States and Europe. J Clin Endocrinol Metab. 2017;102(4):1161‐1173. 28324103 10.1210/jc.2016-2935 PMC5460736

[6] Rosner W, Auchus RJ, Azziz R, Sluss PM, Raff H. Position statement: utility, limitations, and pitfalls in measuring testosterone: an Endocrine Society position statement. J Clin Endocrinol Metab. 2007;92(2):405‐413. 17090633 10.1210/jc.2006-1864

[7] Rosner W, Vesper H. Toward excellence in testosterone testing: a consensus statement. J Clin Endocrinol Metab. 2010;95(10):4542‐4548. 20926540 10.1210/jc.2010-1314

[8] Vesper HW, Botelho JC. Standardization of testosterone measurements in humans. J Steroid Biochem Mol Biol. 2010;121(3-5):513‐519. 20302935 10.1016/j.jsbmb.2010.03.032

[9] Cao ZT, Botelho JC, Rej R, Vesper H. Accuracy-based proficiency testing for testosterone measurements with immunoassays and liquid chromatography-mass spectrometry. Clin Chim Acta. 2017;469:31‐36. 28288785 10.1016/j.cca.2017.03.010 PMC5695555

[10] Cao ZT, Botelho JC, Rej R, Vesper H, Astles JR. Impact of testosterone assay standardization efforts assessed via accuracy-based proficiency testing. Clin Biochem. 2019;68:37‐43. 30928392 10.1016/j.clinbiochem.2019.03.014

[11] Testosterone II (TSTII) Assay Instruction for Use; 11200389, Rev. 07, 2023-03. Siemens Healthineers; 2024.

[12] Testosterone II (TSTII) Assay Instruction for Use; 11205089, Rev. 06, 2023-06. Siemens Healthineers; 2024.

[13] CDC Clinical Standardization Programs: Hormones Reference Laboratory. Updated 05/14/2024. Accessed September 25, 2025. https://www.cdc.gov/clinical-standardization-programs/php/hormones/hormone-reference-laboratory.html

[14] Australian Government National Measurement Institute Certification Reference Material Certificate of Analysis—M914, 2025. Accessed September 26, 2025.

[15] Danilenko U, Vesper HW, Myers GL, Clapshaw PA, Camara JE, Miller WG. An updated protocol based on CLSI document C37 for preparation of off-the-clot serum from individual units for use alone or to prepare commutable pooled serum reference materials. Clin Chem Lab Med. 2020;58(3):368‐374. 31665109 10.1515/cclm-2019-0732 PMC7153737

[16] Botelho JC, Shacklady C, Cooper HC, et al Isotope-dilution liquid chromatography-tandem mass spectrometry candidate reference method for total testosterone in human serum. Clin Chem. 2013;59(2):372‐380. 23213081 10.1373/clinchem.2012.190934

[17] Participant Protocol for CDC Hormone Standardization (CDC HoST) Program-Total Testosterone (TT). Updated 4/26/23, 2025. Accessed September 25, 2025. https://www.cdc.gov/clinical-standardization-programs/media/pdfs/2024/04/Testosterone-Protocol-508.pdf

[18] CDC Hormones Certified Assays and Participants, 2025. Accessed October 22, 2025. https://www.cdc.gov/clinical-standardization-programs/php/hormones/list-of-hormone-certified-assays.html

[19] Tom Fiers JMK . ISSAM: Free & Bioavailable Testosterone calculator, 2025. Accessed September 15, 2025. https://www.issam.ch/freetesto.htm

[20] Vermeulen A, Verdonck L, Kaufman JM. A critical evaluation of simple methods for the estimation of free testosterone in serum. J Clin Endocrinol Metab. 1999;84(10):3666‐3672. 10523012 10.1210/jcem.84.10.6079

[21] Siemens Healthcare Diagnostics . Atellica Testosterone II (TSTII) Assay Instructions for use. 11205089_EN Rev. 07. Siemens Healthineers; 2024-05.

[22] Data Innovations: Total Allowable Error Table. Updated 06/2024, 2024. Accessed September 26, 2024. https://datainnovations.com/allowable-total-error-table

[23] Supplemental Table 1, Atellica TSTII results for TT at concentrations below 9 ng/dL. http://medicalaffairs.siemens-healthineers.com/literature-education/evaluation-siemens-atellica-tstii-testosterone-assay-cdc-host-tt-program-supplemental

[24] CDC Hormone Standardization Program (CDC HoSt): Certified Total Testosterone Assays. https://www.cdc.gov/clinical-standardization-programs/media/pdfs/2024/04/CDC-Certified-Testosterone-Assays-508.pdf

[25] Vesper HW, Bhasin S, Wang C, et al Interlaboratory comparison study of serum total testosterone [corrected] measurements performed by mass spectrometry methods. Steroids. 2009;74(6):498‐503. 19428438 10.1016/j.steroids.2009.01.004

